# Understanding Mosquito Faunal Diversity: An Approach to Assess the Burden of Vector-Borne Diseases in Three Representative Topographies (Rural, Urban, and Peri-Urban) of Ganjam District in Odisha State, India

**DOI:** 10.1155/2024/9701356

**Published:** 2024-09-28

**Authors:** Deepika Panda, Rabi Sankar Pandit, Bijayalaxmi Sahu, Raghavendra Kamaraju, Tapan Kumar Barik

**Affiliations:** ^1^ Department of Zoology Berhampur University, Bhanja Bihar 760007, Odisha, India; ^2^ Integrated Disease Surveillance Programme (IDSP) State Surveillance Unit, Bhubaneswar, Odisha 751001, India; ^3^ ICMR-National Institute of Malaria Research, Sector–8, Dwarka, New Delhi 110077, India

## Abstract

Mosquitoes are the best-known disease vectors for most vector-borne diseases that significantly impact global health in terms of morbidity and mortality. In a geographical area, mosquito faunal diversity often alters with changing climatic factors and variable breeding habitats that differ across seasons. Using biodiversity indicators as tools, a study was conducted in rural, peri-urban, and urban areas of district Ganjam, Odisha state, to determine mosquito faunal diversity as an approach to forecast the possible risk of disease transmission in the three representative topographies. A two-year study was undertaken to assess the alpha diversity of mosquito species by the numerical strength of the species using various eco-diversity indices. Species richness and abundance of mosquito species are significantly higher in peri-urban areas compared to urban and rural areas. The species dominance of *Culex quinquefasciatus* was observed in all three topographies, while *Aedes aegypti*, *Aedes albopictus*, and *Anopheles stephensi* were in urban areas. Species richness may dilute the risk of disease in an area, but increased species dominance, mostly vector species, in a new habitat often allows pathogens to infect newer communities at risk, leading to the emergence of new diseases. The current study indicates the possible risk of lymphatic filariasis (LF) infection in all three topographies. On the other hand, the risk of malaria and dengue/chikungunya transmission is higher in urban areas. With routine entomological monitoring, including vector incrimination, the biodiversity indicators will be the best tool to forecast the risk of vector-borne diseases in an area; accordingly, judicious vector control strategies can be adopted.

## 1. Introduction

Every year, around 7 lakh population suffer from various vector borne diseases (VBDs), contributing to approximately 17% of global infectious diseases [1]. Distribution of such VBDs is often attributed to increased globalization, rapid unplanned and uncontrolled urbanization, population explosion, migration, changing agricultural practice, climate, and biodiversity [2, 3]. As the distribution of vectors often reciprocates with changing environment, this frequently modulates the disappearance or re-emergence of VBDs in an area [4, 5]. Furthermore, shifts in ecological conditions affect the distribution of vectors, leading to changes in disease incidence and their outcomes [6]. Mosquitoes, as key vectors for vector-borne (zoonotic) diseases, significantly affect global health through morbidity (chronic suffering, disability, and social stigma) and mortality. These mosquitoes typically thrive and proliferate in specific and well-defined geographical areas. The spatial shift in these insects boosts species richness in a new area which in turn leads to the emergence of VBD in that area [7–9]. Rising population density, poverty, inadequate health-seeking behaviours, and poor sanitation frequently change the environment, leading to a higher risk of disease transmission in urban and peri-urban areas [10].

Urbanization not only reduces the biodiversity and richness of sylvatic mosquito species but also increases the abundance of species that can adapt to urban ecological niches, increasing the potential risk of human disease transmission, may support novel pathogens to infect human populations and spread of new diseases [8, 11–13]. Changes in vector bionomics, abundance, or distribution can trigger the emergence and spread of new diseases in rural, peri-urban, and urban areas [14].

Mosquitoes are well-known vectors for pathogens that pose a significant threat to the health of both humans and animals globally [15]. Due to tropical climatic conditions, India ranks fifth concerning mosquito faunal diversity [16]. Temperature, humidity, and the availability of breeding sites are key environmental factors that influence mosquito species composition and abundance [17, 18]. Additionally, seasonality directly impacts species abundance, which in turn influences disease transmission [19].

In India, six major vector-borne diseases, malaria, dengue, chikungunya, lymphatic filariasis (LF), Japanese encephalitis (JE), and visceral leishmaniasis (VL) are endemic to the country. Even though the nation will have to eradicate two vector-borne diseases (VBDs) by 2027 such as malaria [20] and lymphatic filariasis [21], the ongoing climatic changes and corresponding modifications to the bionomics of the predominant vector frequently cause the disease pattern to shift from “seasonal to perennial,” with sporadic resurgence and occasional outbreaks. Loss of biodiversity increases disease transmission, whereas regions rich in biodiversity act as a source pool for the entry of new pathogens [22].

Odisha has emerged as the state with the highest number of malaria cases in 2023, with nearly 41,973 occurrences, according to the National Centre for Vector Borne Diseases Control [23]. The reported occurrence of dengue from seasonal to perennial and sporadic cases of JE from endemic districts with microfilaria morbidity in many districts highlights the presence of multiple mosquito vector species with varied bionomics, climatic changes, insecticide use for vector control and due to migration of people.

The present study was carried out for a period of two years in Ganjam district, Odisha, with an aim to assess the biodiversity indicators like species composition, distribution, and relative abundance of mosquitoes, including vector species sampled from three major topographies in the district, to forecast any possible risk of disease transmission, strategize appropriate vector control measures and response to mitigate any impending outbreak.

## 2. Materials and Methods

**Figure 1:**
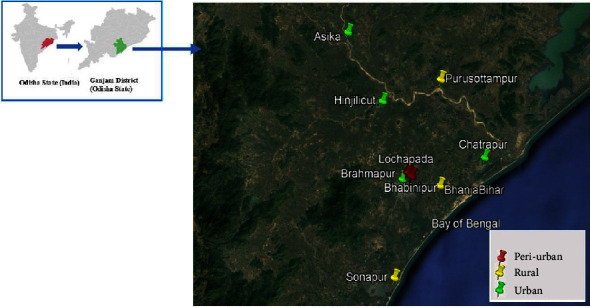
Location of Odisha state in India showing the studied sites in district of Ganjam.

The study was carried out for 24 months from January 2018 to December 2019 in three different topographies: urban (Berhampur: 19.1853°N 84.4738°E, Chhatrapur: 19.2134°N 84.5919°E, Asika: 19.3657°N 84.3953°E, Hinjilicut: 19.2829°N 84.4450°E), peri-urban (Bhabinipur: 19.1916°N 84.4858°E, Lochapada: 19.1947°N 84.4821°E), and rural areas (Purusottampur: 19.3124°N 84.537°E, Sonapur: 19.648°N 84.4631°E, Bhanja Bihar: 19.186°N 84.5258°E) of the Ganjam district bordering north to Andhra Pradesh and off-shore on the east coast of the Bay of Bengal, covering geographical areas of 8070.60 sq. km situated on 19.5860° North Latitude, and 84.6897° East Longitude. Geographically, the district has two-zone, eastern-coastal plain areas while western-hilly and tableland with an extension of the Eastern Ghat to the western region (Figure 1). The district experiences on average 1295.6 mm of annual rainfall. The rainfall generally increases from the coast towards the interior hilly areas. This district experiences up to 32.2°C temperature increases, with a mean daily minimum of 26.9°C. After the end of September, temperature decreases progressively, the drop in night temperature being more rapid. December is the coldest month with the mean daily minimum temperature at 16.6°C and mean daily maximum at 27.5°C. Relative humidities are high about 75 percent throughout the year [24]. The combination of elevated humidity and stagnant water bodies in Ganjam district creates ideal breeding conditions for mosquitoes throughout the year, particularly in coastal areas. Consequently, mosquito species diversity is notably high in this region. Over the past three decades, various ecological changes, including extensive deforestation, frequent cyclones, and widespread insecticide use, have led to an increase in malaria cases [25]. However, no studies have been conducted on mosquito populations in this context.

### 2.1. Collection and Rearing of Mosquitoes

The mosquitoes were sampled at random from three topographies. The prevalent breeding sites for collecting *Anopheles* larvae were ditches, cement tanks, and discarded ceramic basins. Similarly, *Culex* larvae were collected from sewer ditches, swamps, gutters and immatures of *Aedes* mosquitoes were collected from discarded tyres, coconut shells, earthen pots, cement pots, disposed cans, and tree stumps. All the immatures were collected using dropper and dipper. Likewise, adult female *Anopheles* and *Culex* mosquitoes were collected between 5 and 6.30 am and 6 to 8 pm from indoor human dwellings and cattle sheds with the help of a flashlight, oral aspirator and mechanical aspirator. In contrast, adult *Aedes* mosquitoes were collected between 3 and 5 pm from indoor human dwellings and outdoors around vegetation. The adult mosquitoes were transported to the Medical Entomology Laboratory, Dept. of Zoology, Berhampur University, Bhanja Bihar, Odisha, for rearing. Larvae were reared for adult emergence and identified to species based on species-specific morphological characteristics [26–29]. The mosquito colonies were maintained in an insectary at a photoperiod of 12 hours (light)-12 hours (dark), a temperature of 27 ± 2°C with a relative humidity of 75 ± 5%. Adult mosquitoes were fed using 10% glucose solution soaked in a cotton swab and soaked raisins kept in a Petri-dish inside the Barraud cage [30]. The identified adult mosquitoes were stored at −20°C for molecular identification.

### 2.2. Genomic DNA Extraction and Quantification

DNA was isolated by the Bender Buffer method from individual adult mosquitoes [31]. Qualitative and quantitative measurements of isolated DNA, were carried out using 1% Agarose Gel Electrophoresis and a Nanodrop spectrophotometer (Thermo Scientific, U.S.A).

### 2.3. Gene Amplification and Sequencing

The mitochondrial COI gene was amplified by Polymerase chain reaction with published universal primers [32], Forward-LCO1490 5′-GGTCAACAAATCATAAAGATATTGG-3′ and Reverse-HCO21985′-TAAACTTCAGGGTGACCAAAAAATCA-3′. The reaction mixture of COI gene amplification contained 1X PCR buffer, 0.5UTaq DNA, 2.5 mM MgCl_2_, 200 *µ*M dNTPs, 10 pmol of each primer, 100 pmol template DNA, and total dilution was made up to 25 *µ*l. The thermo-cycling program was initial denaturation at 95°C for 5 min followed by 35 cycles of 95°C for 30 sec, 45°C–55°C for 30 sec, and 72°C for 1 min and a final extension step of 7 min at 72°C. The amplicons were resolved by 1.5% agarose gel electrophoresis. The PCR products were sequenced commercially by the Sanger method using ABI Prism 3730XL Big Dye Terminator V3.1 cycle sequencer (Applied Biosystem, USA). The generated nucleotide sequences from each species were compared with available barcode sequences on NCBI using the BLASTn tool.

### 2.4. Data Analysis

The data were subjected to analysis of variance (ANOVA) to test the significance of the data. Further, the Alpha diversity of mosquito species was assessed by the numerical strength of the species in the studied area by using various diversity indices. All the indices were calculated in the Online Biodiversity calculator of AL Young Studios [33].

#### 2.4.1. Simpson's Index of Diversity (D) [34]

(1) $$\begin{array}{c} D=\frac{\sum_{i}n_{i}\left(n_{i}-1\right)}{N\left(N-1\right)}, \end{array}$$

where *n_i_* = the total number of organisms that belongs to species and *N* = the total number of organisms of all species.

The value of *D* ranges between 0 and 1, where 0 represents infinite diversity (all taxa are equally present) and 1, no diversity, which means the bigger the value of *D* the lower the diversity. This is neither intuitive nor logical, so the formula has been modified as 1 − *D* (Simpson's index of diversity or Dominance index) and 1/*D* (Reciprocal index).

#### 2.4.2. Simpson's Index of Diversity or Dominance Index = 1 − D

Here, the value again ranges from 0 to 1. However, now 0 represents no diversity and 1 represents infinity diversity, meaning the higher the value of *D*, the more the diversity.

#### 2.4.3. Reciprocal Index = 1/*D*

Here, the value starts with 1, which means only one species in that community. As the value increases, it represents higher diversity.

#### 2.4.4. Shannon Diversity Index

Shannon index of diversity (*H*′) [35, 36]. It is a diversity index taking into account the number of individuals as well as no. of taxa. Varies from 0 for communities with only a single taxon to high values for communities with many taxa, each with few individuals.

(2) $$\begin{array}{c} H^{\prime }=-\underset{i}{\sum }\left(\frac{n_{i}}{N}\cdot \ln\left(\frac{n_{i}}{N}\right)\right), \end{array}$$

where *n*_*i*_ = number of individuals in the *i*th species and *N* = total no. of entities in the dataset.

As the value of *H*′ increases, the diversity increases.

#### 2.4.5. Evenness of Species

Evenness of species refers to distribution of abundance among the species in a particular community or area of habitat. Pielou's Evenness index (*e*) was used to calculate the evenness of species [37]. It suggests the distribution of individual organisms among the species.

(3) $$\begin{array}{c} e=\frac{H^{\prime }}{H_{\max}}, \end{array}$$

where *H*′ = Shannon Index *H*_max_ = ln (S) = maximum diversity possible *S* = total number of species = species richness.

#### 2.4.6. Species Richness Index

Here, Margalef's index of richness “Dmg” was used [38]. The value can be achieved by dividing the number of species in a sample by the natural log of the number of organisms collected. The number of species per sample is an indicator of richness. When more species are present, richer is the sample or vice-versa.

(4) $$\begin{array}{c} \text{Dmg}=\frac{S-1}{\ln\;N}, \end{array}$$

where *S* = no. of species in the sample and *N* = total no. of species.

#### 2.4.7. Gini Coefficient and Lorenz Curve [33, 39]

(5) $$\begin{array}{c} G=\frac{2\sum_{i}in_{i}}{n\sum_{i}n_{i}}-\frac{N+1}{N}, \end{array}$$

*n*_*i*_ = number of individuals in the *i*th species and *N* = total no. of entities in the dataset.

The dispersion in the species distribution was examined using the Lorenz curve and the Gini coefficient, which is a ratio between 0 and 1. The Lorenz curve used the cumulative percentage of species on the *X*-axis and the cumulative percentage of abundance on the *Y*-axis. Higher equality, or being less scattered from the mean value species distribution, is indicated by a lower Gini coefficient value, and vice versa.

## 3. Results

### 3.1. Molecular Identification of Species

The sequences of the amplicon products were compared with the published sequences to 98–100% similarity. The molecular results of the study were confirmed with the identified morphology-based identifications.

**Figure 2:**
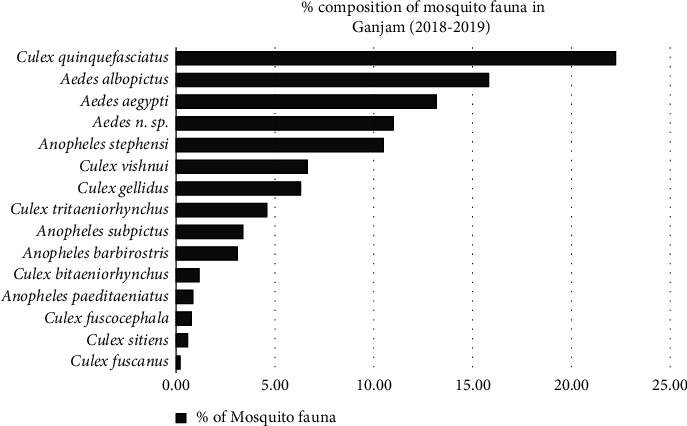
Composition of mosquito fauna (%) in three different topographies (rural, peri-urban, and urban), Ganjam district of Odisha, Eastern India (2018-2019).

During the two years of monitoring, a total of 2534 mosquitoes (896 adult females and 1638 larvae) belong to three genera, *Anopheles*, *Culex,* and *Aedes*. Fifteen (15) different species were collected from the three topographies: peri-urban, urban, and rural. Of these, 426 adults and 700 larvae were recorded from peri-urban, 218 adults and 844 larvae from urban and 252 adults and 94 larvae were recorded from rural. From the total 15 species identified from three genera, 15 were found in peri-urban topography, 10 species in rural, and 9 in urban topography. Of the total collected samples (both larvae and adult), 42.42% belonged to *Culex* species, 39.9% to *Aedes* species and 17.68% were to *Anopheline* species. From the *Culex* samples, eight different species were identified of which a high percentage were *Cx. quinquefasciatus* (52.37%) followed by *Cx. vishnui* (15.72%) and *Cx. gelidus* (14.79%), whereas *Cx. fuscanu*s with low percentage (0.47%). Among anophelines, *An. stephensi* was found in a very high number (59.15%) followed by *An. subpictus* (18.97%), *An. barbirostris* (17.41%), and significantly low proportion of *An. paeditaeniatus* (4.46%). The three different *Aedes* species that were collected almost showed an equal proportion of three species, *Ae. aegypti* (32.94%), *Ae. albopictus* (39.56%), and *Ae. n. species* (27.50%) (Unpublished) (Figure 2). The above results showed a significantly higher distribution of genera *Aedes* in all three eco-types. Of the total collected samples, there are seven (7) known major vectors of arboviruses (*Cx. tritaenorhynchus*, *Cx. bitaenorhynchus*, *Cx. gelidus*, *Cx. fuscocephala*, *Cx. vishnui*, *Ae. aegypti*, and *Ae. albopictus*) besides few secondary vectors for JE (*Cx. quinquefasciatus*, *An. subpictus* and *An. barbirostris*) in India [40]. *Ae. aegypti* was found predominant in urban areas while the earliest known sylvatic species, *Ae. albopictus*, was later found to be predominant in both urban and peri-urban areas [41] and also in the present study, which defines the paradigm shift of *Ae. albopictus* as an invasive species from rural to establish well in new ecological set-up of urban and peri-urban areas [5]. Apart from the well-known filarial vector *Cx. quinquefasciatus*, which was found to be predominant in urban areas, the two arboviral disease vectors *Cx. vishnui* and *Cx. gelidus* were found to be predominant in the peri-urban and urban set-ups, respectively. Whereas major urban malaria vector species, *An. stephensi* is localized and predominant in the urban set-up (66.69%). Of the total samples, 48.38% of mosquitoes are known to be implicated in the potential transmission of arboviral diseases like dengue (28.92%) and JE (26.67%), while 10.45% and 22.21% of mosquito vectors for potential transmission of malaria and lymphatic filariasis, respectively.

### 3.2. Species Richness

**Figure 3:**
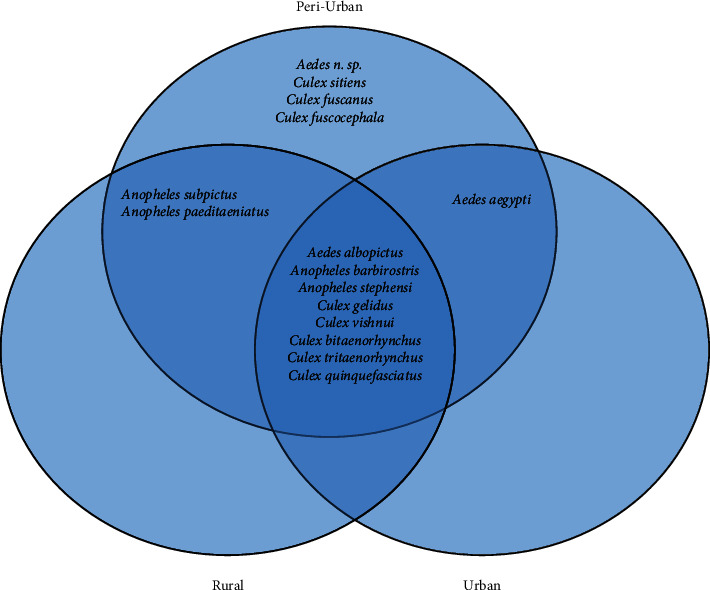
Distribution of mosquito fauna in three different topographies.

The species richness (Species richness refers to the number of species in a defined area) was found to be high in the peri-urban area, followed by the rural area. In contrast, the species abundance (Total number of individuals of a given species within a specific region is referred to as Species abundance) was significantly lower in the rural area compared to peri-urban and urban areas. However, there was no significant difference (*p* > 0.05) in the abundance of mosquito species in the three topographies (*p*=0.1681 (*p* > 0.05)). *Cx*. *quinquefasciatus* predominant in all three topographies (22.22%), followed by *Ae. albopicuts* (15.79%) and *Ae. aegypti* (13.14%) while the species richness of *Cx. fuscanus* was low (0.20%). Species richness of *An. subpictus* was found to be high in rural followed by peri-urban areas, as indicated in (Figure 3). Similarly, species like *Cx. fuscocephala*, *Cx. sitiens*, *Cx. fuscanus*, and *Ae. n. sp.* (Unpublished), only confined to the peri-urban area. Meanwhile, the relative abundance of both species *An. paeditaeniatus* and *An. subpictus* gradually decreased from rural to peri-urban topographies (Figure 3).

### 3.3. Simpson Index (D)

In all three topographies, Simpson's index (*D*) was also found to be low. However, it is significantly low in peri-urban with a value of *D* = 0.1402 compared to both rural and urban areas showing an almost similar range of *D* = 0.1704 and *D* = 0.1752 (Table 1).

### 3.4. Simpson Dominance Index

In all three topographies, the Simpson Dominance index was found in the ratio of rural : peri-urban : urban (0.8276 : 0.8588 : 0.7788), respectively (Table 1).

### 3.5. Reciprocal Index

The reciprocal Simpson index was found to be higher in the peri-urban topography with a value of 7.135 while almost similar though comparatively low in both urban and rural topographies with values, i.e., 4.521 and 5.821. Thus, the result shows high peri-urban diversity compared to rural and urban topographies (Table 1).

### 3.6. Margalef Richness Index

This Margalef richness index also shows a high value in peri-urban (1.992), followed by rural (1.539) and low in the urban topography (1.148) (Table 1).

### 3.7. Shannon Index

In the peri-urban topography, the Shannon index was found to be significantly high (H-2.204), followed by rural (H-1.968), while less in the urban set-up (H-1.671) (Table 1).

### 3.8. Species Evenness

However, the species evenness is almost equal in both rural (0.854) and peri-urban (0.813) set-ups and higher in comparison to the urban topography (0.760) (Table 1).

### 3.9. Gini Coefficient

**Figure 4:**
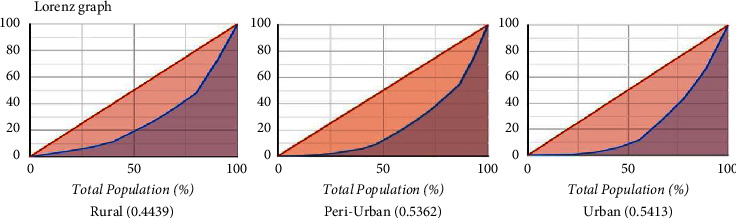
Lorenz curve showing diversity of mosquito species in three different topographies.

The Gini coefficient is a crucial index for ecological studies and infectious diseases [42, 43]. Whereas the Lorenz Curve is a tool to measure the inequality and is a graphical representation of distribution against the uniform distribution (Figure 4). The coefficient ranges from 0 (or 0%) to 1 (or 100%), with 0 representing perfect equality and 1 representing perfect inequality. The present study, in three different topographies yields the Gini coefficient (*G*) values of peri-urban (0.5362), urban (0.5413), and rural (0.4439), respectively. The diversity of mosquito populations in the rural topography showed less diversity than in the peri-urban and urban topographies. In contrast, an almost similar pattern of high diversity was observed in both peri-urban and urban topographies (Table 1).

## 4. Discussion

The interplay of the habitat type, climatic conditions, and seasonal change significantly influenced mosquito species richness. The presence of genus *Anopheles, Culex,* and *Aedes* mosquitoes in study areas such as rural, peri-urban, and urban topographies during the study period in Ganjam district, Odisha, indicates a potential risk of mosquito-borne diseases and highlights the vulnerability of local inhabitants as stated by Mwangangi et al. in a study in Malindi along the Kenya coast [10]. Though high in the urban set-up, the relative abundance of *Cx. quinquefasciatus* in all three topographies entails itself as a predominant species, indicating the increased risk of potential transmission of lymphatic filariasis (LF) [44–46]. Behavioural plasticity suggests a need for vector control measures to prevent profuse breeding and proliferation in urban topography [47]. About 55 species of mosquitoes under 12 genera were reported from coastal Odisha [48]. A new *Culex* mosquito species, *Cx. singhbhumensis* was also reported in Orissa [49]. The current study reveals that high species diversity (*p* value) in peri-urban topography increases the risk of disease emergence, contradicting the “dilution-effect” model, which states that uncertainty about the risk of transmission, regardless of the diversity of the vector, reduces the risk of amplification and disease spread [50]. For this reason, the current study is necessary to clarify the species richness, abundance, and distribution of vectors in each of the three configurations of the heterogenic human population. It has been reported that increased species richness can amplify disease transmission as vector-borne diseases necessitate multiple mosquito species to influence disease transmission rate [51, 52]. The high species richness in both peri-urban and urban topographies, consistent with Abella-Medrano et al. findings, indicates an increased risk of filariasis due to factors like rural activity, poor sanitation, and increasing population density [53]. The abundance of three species, *Ae. aegypti*, *Ae. albopictus*, and *An. stephensi*, as high in urban topography compared to other species, substantiates the increased adaptation to the urban environment and through a wide range of breeding sources and man-made habitat, thereby increasing the threat of diseases like dengue and urban malaria transmission [53]. Due to changing climate and anthropogenic activities also contribute to changes in their adaptation [54]. Hence, the species composition of the mosquito population also depends on the type of environment [55]. The diversity of mosquito breeding habitats supports oviposition and proliferation in mosquito species, which are crucial for vector monitoring and control strategies [56–59]. Diversity indices and species richness estimates show that the mosquito fauna was more diverse in urban areas, with greater diversity in peri-urban areas and less diversity in rural settings.

Simpson's index (D), a widely recognized measure of biodiversity, consistently indicated low values across all three study areas. Notably, the peri-urban topography exhibited the lowest Simpson's index (*D*: 0.1402), suggesting a higher level of species diversity (1126) in comparison to both rural (346) and urban areas (1062), which indicates that the peri-urban set-up harbours a more abundant and diverse mosquito community. The reciprocal Simpson index supported these findings, reinforcing the notion of increased species diversity in the peri-urban topography. Furthermore, the Shannon index, a fundamental metric of species diversity, showed significantly higher values in the peri-urban set-up (2.204), followed by rural (1.968) and comparatively lower values (1.671) in urban topographies. These results further emphasize the ecological significance of the peri-urban topography, where mosquito species coexist more balanced and diversely. The assessment of species evenness, an indicator of species distribution equilibrium, revealed relatively similar values in both rural (0.854) and peri-urban (0.813) topographies, with higher evenness compared to the urban (0.760) areas. This highlights the relatively equitable distribution of mosquito species in rural and peri-urban topographies, further supporting their higher biodiversity. The Gini coefficient, a crucial ecological and epidemiological tool, and the Lorenz curve were employed to assess the degree of inequality in mosquito species distribution within the studied areas. The Gini coefficient values for the peri-urban (0.5362) and urban (0.5413) topographies indicated higher inequality in mosquito populations compared to the rural (0.4439) area. This suggests a more even distribution of mosquito species in rural topography. Overall, these biodiversity indices shed light on the diversity, dominance, and evenness of mosquito populations in each of the three distinct geographical areas: peri-urban, urban, and rural. They offer valuable insights into the ecological dynamics of these studied regions. These findings highlight a significant contrast in mosquito species diversity and distribution among the peri-urban, urban, and rural landscapes. The peri-urban region emerges as an ecological hotspot, exhibiting greater richness, diversity, and evenness of mosquito species. These observations have potential implications for understanding mosquito-borne infectious diseases in different environments, calling for targeted disease surveillance and control strategies. Further research is warranted to comprehend the intricate dynamics of mosquito populations and their ecological significance in these topographies.

The Lorenz curve, a visual representation of species abundance distribution across habitats, indicates higher abundance of mosquito species in peri-urban and urban areas compared to rural topographies. The curve plots cumulative percentages of species abundance against the total number of species observed. Here, the diagonal line shows the line of equality (Red line); however, the area between the curve (Blue line) and the line of equality shows the distribution of species abundance across different habitats. This helps in biodiversity studies in identifying areas with high species diversity and areas needing more attention against disease-causing mosquito vectors.

The richness and species abundances in peri-urban topography, with the dominance of vector species like *Ae. albopictus*, *Cx. vishnui*, *Cx. quiquefasciatus*, and *An. stephensi* in urban area suggests the risk of transmission and outbreak of such arboviral and protozoal diseases, indicating a stronger correlation between disease and vector distribution and outbreak [60]. The presence of *Cx. vishnui*, a principal vector of JE, and the sylvatic species like a vector of dengue/chikungunya in these topographies may exacerbate the risk of outbreaks and impact human health [46, 61]. Anthropogenic behavior and host-seeking behavior contribute to species movement and its consequential disease implications. These factors also create opportunities for different mosquito species such as new species and invasive species to establish in a particular eco-type.

## 5. Conclusion

VBDs share a significant part of endemic diseases. Change in vector bionomics, vector abundance, composition, or distribution in a new area enhances the opportunity for the human-vector interface, triggering the emergence of diseases and their spread in that topography. Research so far has focused on distribution rather than related indices explaining transmission risk. The present study was undertaken to determine the distribution of mosquito species in relation to the vectors that are found in three different topographies of the Ganjam district of Odisha. The current study assessed the biodiversity indicators of vector species and showed the possibility of risk of infection of lymphatic filariasis in all three topographies while the risk of dengue/chikungunya and malaria in urban areas. With routine vector monitoring, including longitudinal and seasonal vector incrimination studies in these topographies and indices, will be a tool to forecast the risk of VBDs and for adequate control.

## Figures and Tables

**Table 1:** Biodiversity Indices of mosquito fauna in rural, peri-urban, and urban areas of Ganjam district of Odisha state (2018-2019).

Sl. no.	Indices-biodiversity	Rural	Peri-urban	Urban
1	Total number of species (species richness)	10	15	9
2	Total number of organisms (species abundance)	346	1126	1062
3	Simpson's index	0.1704	0.1402	0.1752
4	Simpson's dominance index	0.8276	0.8588	0.7788
5	Reciprocal Simpson's index	5.821	7.135	4.521
6	Shannon index	1.968	2.204	1.671
7	Gini coefficient	0.4439	0.5362	0.5413
8	Margalef richness index	1.539	1.992	1.148
9	Evenness	0.854	0.813	0.760

## Data Availability

The data presented in this study are available from the corresponding author upon reasonable request.

## References

[1] World Health Organization WHO (2020). Vector borne diseases fact sheet. https://www.who.int/news-room/fact-sheets/detail/vector-borne-diseases#:%7E:text=Vector%2Dbornediseasesaccountfor,infectiontransmittedbyAnophelinemosquitoes.

[2] Neiderud C. J. (2015). How urbanization affects the epidemiology of emerging infectious diseases. *Infection Ecology and Epidemiology*.

[3] Hoberg E. P., Brooks D. R. (2015). Evolution in action: Climate change, biodiversity dynamics and emerging infectious disease. *Philosophical Transactions of the Royal Society B: Biological Sciences*.

[4] Engering A., Hogerwerf L., Slingenbergh J. (2013). Pathogen–host–environment interplay and disease emergence. *Emerging Microbes and Infections*.

[5] Muller R., Reuss F., Kendrovski V., Montag D., Marselle M., Stadler J., Korn H., Irvine K., Bonn A. (2019). Vector-borne diseases. *Biodiversity and Health in the Face of Climate Change*.

[6] Rocklöv J., Dubrow R. (2020). Climate change: An enduring challenge for vector-borne disease prevention and control. *Nature Immunology*.

[7] World Health Organization WHO (2014). *A Global Brief on Vector-Borne Diseases*.

[8] Lafferty K. D. (2009). The ecology of climate change and infectious diseases. *Ecology*.

[9] Dhimal M., Ahrens B., Kuch U. (2015). Climate change and spatiotemporal distributions of vector-borne diseases in Nepal – a systematic synthesis of literature. *PLoS One*.

[10] Mwangangi J. M., Midega J. T., Kahindi S. (2012). Mosquito species abundance and diversity in Malindi, Kenya and their potential implication in pathogen transmission. *Parasitology Research*.

[11] McKinney M. L. (2006). Urbanization as a major cause of biotic homogenization. *Biological Conservation*.

[12] Medeiros-Sousa A. R., Fernandes A., Ceretti-Junior W., Wilke A. B. B., Marrelli M. T. (2017). Mosquitoes in urban green spaces: Using an island biogeographic approach to identify drivers of species richness and composition. *Scientific Reports*.

[13] Lee K. S., Divis P. C., Zakaria S. K. (2011). *Plasmodium knowlesi*: Reservoir hosts and tracking the emergence in humans and macaques. *PLoS Pathogens*.

[14] World Health Organization WHO (2015). Connecting Global Priorities: Biodiversity and Human Health: A state of knowledge review. https://www.who.int/publications/i/item/connecting-global-priorities-biodiversity-and-human-health.

[15] Mayi M. P., Bamou R., Djiappi-Tchamen B. (2020). Habitat and seasonality affect mosquito community composition in the west region of Cameroon. *Insects*.

[16] Foley D. H., Rueda L. M., Wilkerson R. C. (2007). Insight into global mosquito biogeography from country species records. *Journal of Medical Entomology*.

[17] Beck-Johnson L., Nelson W., Paaijmans K., Read A., Thomas M., Bjornstad O. (2017). The importance of temperature fluctuations in understanding mosquito population dynamics and malaria risk. *Royal Society Open Science*.

[18] Grillet M. E. (2000). Factors associated with the distribution of *Anopheles aquasalis* and *Anopheles oswaldoi* (Diptera: Culicidae) in a malarious area, north-eastern Venezuela. *Journal of Medical Entomology*.

[19] Preechaporn W., Jaroensutasinee M., Jaroensutasinee K. (2007). Seasonal prevalence of *Aedes aegypti* and *Ae. albopictus* in three topographical areas of southern Thailand. *World Academy of Science, Engineering and Technology*.

[20] Directorate National Vector Borne Disease Control Programme (2016). *National Framework for Malaria Elimination in India (2016–2030)*.

[21] Ministry of Health and Family Welfare (2017). National health policy. https://main.mohfw.gov.in/sites/default/files/9147562941489753121.pdf.

[22] Keesing F., Belden L., Daszak P. (2010). Impacts of biodiversity on the emergence and transmission of infectious diseases. *Nature*.

[23] (2024). National center for vector borne diseases control (NCVBDC). https://ncvbdc.mohfw.gov.in/WriteReadData/l892s/833563291720527717.pdf.

[24] Odisha district gazetteers Ganjam: climatic condition. https://ganjam.odisha.gov.in/sites/default/files/2023-06/2018032995.pdf.

[25] Dash S. (2014). Morphological and molecular characterization of genus *Anopheles* (DIPTERA: CULICIDAE) of Ganjam district Orissa, India. *Records of the Zoological Survey of India*.

[26] Christophers S. R. (1933). *Family Culicidae Tribes Anophelini: The Fauna of British India, Including Ceylon and Burma- Diptera*.

[27] Barraud P. J. (1934). *The Fauna of British India Including Ceylon and Burma. Diptera Vol. V. Family Culicidae Tribes Megarhinini and Culicini*.

[28] Reuben R., Tewari S. C., Hiriyan J., Akiyama J. (1994). Illustrated keys to species of culzx (*Culex*) associated with Japanese encephalitis in Southeast Asia (Diptera: Culicidae). *Mosquito Systematics*.

[29] Tyagi B. K., Munirathinam A., Venkatesh A. (2015). A catalogue of Indian mosquitoes. *International Journal of Mosquito Research*.

[30] Raghavendra K., Barik T. K., Adak T. (2010). Development of larval thermotolerance and its impact on adult susceptibility to malathion insecticide and *Plasmodium vivax* infection in *Anopheles stephensi*. *Parasitology Research*.

[31] Collins F. H., Mendez M. A., Rasmussen M. O., Mehaffey P. C., Besansky N. J., Finnerty V. (1987). A Ribosomal RNA gene probe differentiates member species of the *Anopheles gambiae* complex. *The American Journal of Tropical Medicine and Hygiene*.

[32] Folmer O., Black M., Hoeh W., Lutz R., Vrijenhoek R. (1994). DNA primers for amplification of mitochondrial cytochrome c oxidase subunit I from diverse metazoan invertebrates. *Molecular Marine Biology and Biotechnology*.

[33] Young T. M. (2017). Biodiversity calculator for the Simpson and Shannon index. https://www.alyoung.com/labs/biodiversity_calculator.html.

[34] Simpson E. H. (1949). Measurement of diversity. *Nature*.

[35] Shannon C. E., Weaver W. (1949). *A Mathematical Theory of Communication*.

[36] Wolda H. (1983). Diversity, diversity indices and tropical cockroaches. *Oecologia*.

[37] Pielou E. C. (1966). The measurement of diversity in different types of biological collections. *Journal of Theoretical Biology*.

[38] Margalef R. (1958). Information theory in ecology. *General Systems*.

[39] Sun T., Zhang H., Wang Y., Meng X., Wang C. (2010). The application of environmental Gini coefficient (EGC) in allocating waste water discharge permit: the case study of watershed total mass control in Tianjin, China. *Resources, Conservation and Recycling*.

[40] Pearce J. C., Learoyd T. P., Langendorf B. J., Logan J. G. (2018). Japanese encephalitis: the vectors, ecology and potential for expansion. *Journal of Travel Medicine*.

[41] Eapen A., Ravindran K. J., Dash A. P. (2010). Breeding potential of *Aedes albopictus* (Skuse, 1895) in chikungunya affected areas of Kerala, India. *Indian Journal of Medical Research*.

[42] Damgaard C., Weiner J. (2000). Describing inequality in plant size or fecundity. *Ecology*.

[43] Kerani R. P., Handcock M. S., Handsfield H. H., Holmes K. K. (2005). Comparative geographic concentrations of 4 sexually transmitted infections. *American Journal of Public Health*.

[44] Oduola A. O., Awe O. O. (2006). Behavioural biting preference of *Culex quinquefasciatus* in human host in Lagos metropolis Nigeria. *Journal of Vector Borne Diseases*.

[45] Chatterjee S., Azmi S., Das S. (2015). Seasonal prevalence and blood meal analysis of filarial vector *Culex quinquefasciatus* in coastal areas of Digha, West Bengal, India. *Journal of Vector Borne Diseases*.

[46] Panda D., Barik T. K. (2022). Molecular characterization and genetic divergence of seven *Culex* mosquito (Diptera: Culicidae) species using Mt COI gene from Odisha State, India. *The Journal of Basic and Applied Zoology*.

[47] Bhattacharya S., Basu P., Bhattacharya S. C. (2016). The southern house mosquito, *Culex quinquefasciatus*: profile of a smart vector. *Journal of Entomology and Zoology Studies*.

[48] Dash S., Hazra R. K. (2011). Mosquito diversity in the Chilika lake area, Orissa, India. *Tropical Biomedicine*.

[49] Rajavel A., Natarajan R. (2008). Mosquitoes of the mangrove forests of India: Part 7—an overview. *Journal of the American Mosquito Control Association*.

[50] Chaves L. F., Koenraadt C. J. M. (2010). Climate change and highland malaria: Fresh air for a hot debate. *The Quarterly Review of Biology*.

[51] Roche B., Rohani P., Dobson A., Guégan J. F. (2013). The impact of community organization on vector-borne pathogens. *The American Naturalist*.

[52] Rakotonirina A., Maquart P. O., Flamand C., Sokha C., Boyer S. (2023). Mosquito diversity (Diptera: Culicidae) and medical importance in four Cambodian forests. *Parasites & Vectors*.

[53] Abella-Medrano C. A., Ibañez-Bernal S., MacGregor-Fors I., Santiago-Alarcon D. (2015). Spatiotemporal variation of mosquito diversity (Diptera: Culicidae) at places with different land-use types within a neotropical montane cloud forest matrix. *Parasites & Vectors*.

[54] Rao B., George B. (2010). Breeding patterns of *Aedes stegomyia albopictus* in periurban areas of Calicut, Kerala, India. *Southeast Asian Journal of Tropical Medicine and Public Health*.

[55] Kaboré D. P. A., Soma D. D., Gil P. (2023). Mosquito (Diptera: Culicidae) populations in contrasting areas of the western regions of Burkina Faso: species diversity, abundance and their implications for pathogen transmission. *Parasites and Vectors*.

[56] Mattah P., Futagbi G., Amekudzi L. (2017). Diversity in breeding sites and distribution of *Anopheles* mosquitoes in selected urban areas of southern Ghana. *Parasites and Vectors*.

[57] Wilke A., Chase C., Vasquez C. (2019). Urbanization creates diverse aquatic habitats for immature mosquitoes in urban areas. *Scientific Reports*.

[58] Ondiba I. M., Oyieke F. A., Athinya D. K., Nyamongo I. K., Estambale B. B. A. (2019). Larval species diversity, seasonal occurrence and larval habitat preference of mosquitoes transmitting Rift Valley fever and malaria in Baringo County, Kenya. *Parasites & Vectors*.

[59] Rueda L. M. (2008). Global diversity of mosquitoes (Insecta: Diptera: Culicidae) in freshwater. *Developments in Hydrobiology*.

[60] Lutomiah J., Bast J., Clark J. (2013). Abundance, diversity, and distribution of mosquito vectors in selected ecological regions of Kenya: public health implications. *Journal of Vector Ecology*.

[61] World Health Organization WHO (2017). https://www.who.int/india/news/detail/12-07-2017-launch-of-the-national-framework-for-malaria-elimination-in-india-2016-2030.

